# Mutant p53: One, No One, and One Hundred Thousand

**DOI:** 10.3389/fonc.2015.00289

**Published:** 2015-12-21

**Authors:** Dawid Walerych, Kamil Lisek, Giannino Del Sal

**Affiliations:** ^1^Laboratorio Nazionale CIB, Area Science Park Padriciano, Trieste, Italy; ^2^Dipartimento di Scienze della Vita, Università degli Studi di Trieste, Trieste, Italy

**Keywords:** p53 mutation, gain-of-function, cancer, drug therapy, combination, oncogenes, tumor suppressor proteins

## Abstract

Encoded by the mutated variants of the *TP53* tumor suppressor gene, mutant p53 proteins are getting an increased experimental support as active oncoproteins promoting tumor growth and metastasis. p53 missense mutant proteins are losing their wild-type tumor suppressor activity and acquire oncogenic potential, possessing diverse transforming abilities in cell and mouse models. Whether various mutant p53s differ in their oncogenic potential has been a matter of debate. Recent discoveries are starting to uncover the existence of mutant p53 downstream programs that are common to different mutant p53 variants. In this review, we discuss a number of studies on mutant p53, underlining the advantages and disadvantages of alternative experimental approaches that have been used to describe the numerous mutant p53 gain-of-function activities. Therapeutic possibilities are also discussed, taking into account targeting either individual or multiple mutant p53 proteins in human cancer.

## p53 Mutants – Active Oncoproteins

Mutations in the *TP53* gene occur in almost every type of cancer, with frequencies that vary between 10% (hematopoietic malignancies) and 96% (high grade ovarian serous carcinoma) (1). Cancer genome sequencing studies confirm that *TP53* is the most commonly mutated tumor suppressor gene in human cancers (2). The majority of studies indicate that the presence of mutated *TP53* is associated with bad prognosis in various cancer types (3). *TP53* mutations are known first and foremost to inactivate the oncosuppressive properties of the wild-type p53 protein as a transcription factor (loss-of-function – LOF). However, since p53 acts as a tetramer, expressed *TP53* mutant variants can also exert a dominant negative (DN) effect over their wild-type counterpart, and additionally they can arm cancer cells with novel oncogenic gain-of-function (GOF) activities (4–6).

In over 70% of cases, the *TP53* mutations are missense, most frequently within the region encoding the core domain of the p53 protein, which is responsible for binding DNA (7). Although the spectrum of the *TP53* missense mutations is vast – counting about 1,800 different amino-acid changes (8) – several hotspot p53 mutants, in particular, affecting residues R273, R248, R175, and G245 of the p53 protein, are present with a higher frequency both in sporadic tumors (together over 21% of total missense mutations) and in individuals with the Li–Fraumeni syndrome (LFS), a genetic disorder caused by inherited *TP53* mutations that predispose carriers to an early-onset development of various cancers (9).

The hotspot changes in p53 are traditionally classified as “conformational” or “DNA contact” mutations. This notion comes from the biophysical observation that the former group disturbs the proper folding of the core domain of p53, thus depriving it of the ability to bind the DNA and transactivate its target genes, while the latter group is composed of mutations in residues that are responsible for directly binding DNA, with a near-native core domain structure (10, 11). In the LFS, a wild-type *TP53* allele is usually present, whereas in LFS tumors, it is often (in the 40–60% of cases) subjected to inactivation (loss of heterozygosity – LOH) – a process that is observed both in mouse LFS models (12) and in humans (13), involving various mechanisms of wild-type *TP53* inactivation (14). Interestingly, it has been recently noted that in the embryonic stem cells from LFS mice the lost allele is often the mutant one, suggesting that a bi-directional *TP53* LOH process may function as a cell-fate checkpoint and that there exists a selective pressure against the heterozygous *TP53* state (15).

p53 mutant proteins are stabilized and protected from degradation in a tumor microenvironment by various oncogenic signaling pathways (16, 17), and several studies in mutant p53 knock-in (KI) mice showed that the presence of p53 mutants promotes tumor growth with higher metastasis rate and different tissue spectrum than the absence of wild-type p53 (12, 18). These *in vivo* proofs of mutant p53 GOF came as confirmation of the initial observations in cell models that mutant p53 missense variants may actively support cell transformation (19, 20).

Even though the oncogenic activity related to GOF p53 mutants has been described many times in the last 25 years of research on p53, there are still doubts concerning its significance. Current approaches are only starting to resolve whether missense p53 mutants can be regarded as essentially one oncoprotein endowed with a conserved tumorigenic activity, or they represent a population of different oncoproteins, each exerting its unique oncogenic potential. Mutant p53 is still not used in standard clinical practice as a target of anti-cancer therapies. We discuss these issues in the following sections of this review.

## One or Many – “Mutant p53” vs. “p53 Mutants”

The rising importance of the GOF of p53 mutants in cancer has led to numerous studies describing their mechanisms of action and a brought forward question how much the obtained results can be generalized across different mutant p53 variants and cellular or cancer backgrounds.

A minority of these studies is based on mutant p53 KI mouse models and led to a number of discoveries in the field, including (i) the inhibitory role of mutant p53 on MRE11 protein and the induction of genomic instability (human *TP53* KI “HupKI” mouse model) (21), (ii) the transcription-based activation of PDGFRβ signaling in pancreatic cancer model (22), (ii) the transcriptional activation of oncogenic Pla2g16 phospholipase (23), and (iv) the confirmation of prior cell-based reports on a mutant p53-mediated inhibition of the p63/p73 oncosuppressive activity (12, 18). The LFS mouse-model-based studies underlined differences between GOF properties of different p53 mutants and among the consequences of *TP53* mutations in human and in mouse. Comparative studies of the R270H and R172H variants in KI mice showed different tumor spectra confirming the notion that the GOF of p53 mutants may differ (12). These spectra, however, turned out to be different also from the spectra caused by human counterparts of these mutant p53 variants – R273H and R175H – in patients with LFS (e.g., lack of mammary carcinomas in mice – frequent in humans) (9). On the other hand, the investigation in KI mice of the R246S p53 mutant, corresponding to the human R249S p53 hotspot mutant, showed no clear indication of GOF (24), whereas in human cell-based experiments, this variant was demonstrated to induce growth, chemoresistance, and a specific mutant p53 transcriptional program in several studies (25–27). Altogether these results indicate that mouse models – albeit very informative – may have their limitations and require careful confirmation of their significance in human systems.

Most of the human cell line-based studies on mutant p53 are based on initial phenotype-related experiments or large scale analysis, such as gene expression microarray or ChIP sequencing, leading to discovery of mechanisms/targets associated with a particular mutant variant in its endogenous background. In most cases, validation in other mutant p53 variants/backgrounds is also reported. Such studies have led to describing important roles of mutant p53 in direct inhibition of the p63/p73-mediated tumor suppression (28, 29), activation of the cell cycle drivers, such as Cyclins (30, 31), the vitamin D3 receptor signaling (32), steroid synthesis (mevalonate pathway) (33), the ID4-mediated angiogenesis (34), or nucleotide homeostasis (26), to name a few. A comprehensive list of these studies published since 2005 – with the indication of the initially tested mutant p53 variant(s) and p53 mutants used for validation – is shown in Table 1.

**Table 1:** **Selected mutant p53 gain-of-function effects, mediators, and related therapeutic opportunities, published since 2005**.

Mutant p53 discovered	Mutant p53 validated	Pathway(s)	Mediator(s)	Downstream proteins/genes	Leading model(s)	Mutant p53-related phenotype	Suggested treatment	Reference
R248W	R273H, R175H	DNA damage response	MRE11	AKT	HUPKI mice/MEFs	Genomic instability	–	(21)
	R175H, R280K, L194F, R273L, R249S, R248Q, C242F	DNA damage response	ETS2^a^	TDP2^a^	Li–Fraumeni-derived cell line	Chemoresistance	Etoposide	(35)
	R273L, R249S, R280K, R175H	Nucleotide homeostasis	ETS2^a^	Nucleotide metabolism genes^a^	Li–Fraumeni and breast cancer cells	Cell proliferation	–	(26)
R175H	L194F, R273H	IL-8 and GRO-α signaling	NFYA^a^	Cyclin A, B, E, CDK1, CDC25C^a^	Breast cancer cell lines	Cell proliferation	–	(30)
	R273H, R280K	Interleukin signaling,	ID4	IL-8, GRO-α	Breast cancer cell lines	Angiogenesis	–	(34)
	R273H	VDR signaling	VDR^a^	IGFBP3, CYP24A1^a^	Breast cancer cell lines	Reduced apoptosis	Vitamin D3 restriction?	(32)
	R273H, R280K	PDGF receptor β signaling	p73^a^, NFY complex^a^	PDGFRβ^a^	Pancreatic cancer mouse model and cell lines	Metastasis	Imatinib	(22)
	H179R, G245S, R248Q, R249S R273H	Phospholipid metabolism	ETS2^a^	Pla2g16^a^	KI mouse model, osteosarcoma cell line	Tumor growth and metastasis	–	(23)
R280K	R273H	Cell cycle, cell movement	Pin1	Cyclin E2, BUB1, DEPDC1^a^	Breast cancer cell lines	Cell proliferation, migration	Pin1 inhibitors?	(31)
	R175H	TGFβ-induced migration/invasion	SMAD/p63^a^	SHARP-1, Cyclin G2	Breast cancer cell lines	Metastasis	–	(29)
	R175H, R273H, M237I	TNFα-driven inflammation	DAB2IP	JNK, NF-κB, and their targets	Breast cancer cell lines	Cancer-related inflammation	–	(36)
R273H	R280K	Steroid synthesis	SREBP1/2^a^	MVK, FDFT1, TM7SF2, NSDHL^a^	Breast cancer cell lines	Tumor growth	Statins	(33)
	–	HB-EGF signaling	NRD1	–	p53 null lung carcinoma	Invasion	–	(37)
	R280K, L194F	DNA replication, PARP signaling	–	PARP, MCM4, PCNA	Breast cancer cell lines	Cell proliferation	PARP inhibitors?	(38)
R175H, R273H (overexpressed)	R280K	EGFR/integrin signaling	p63	α5β1 integrin, EGFR	p53 null lung carcinoma, breast cancer cell lines	Cell motility, invasion	–	(39)
R175H, R273H, D281G (overexpressed)	–	NF-κB signaling	–	NFKB2^a^	p53 null lung carcinoma	Chemoresistance	Etoposide	(40)
R175H, R248Q, R273H (overexpressed)	R175H, R273H	Glucose metabolism, Warburg effect	RhoA/ROCK	GLUT1	p53 null lung carcinoma, MEFs, breast cancer cell lines	Tumor growth	–	(41)
R175H, R248Q, R248W, R249S, R273H, R282W (overexpressed)	R273H, R280K	Membrane and secreted signaling factors	p63^a^	DKK1, METTL7B, TFPI2^a^	p53 null lung carcinoma, breast cancer cell lines	Invasion	–	(42)
V143A, R175H, R248W, R249S, R273H, R282W (overexpressed)	R175H, R248Q, R273C	Cell cycle, apoptosis	TopBP1^a^, p63/p73^a^, NFY^a^	Cyclin A, B, E, CDK1, CDC25C, BAX, NOXA^a^	p53 null lung carcinoma, breast cancer cell lines	Proliferation	Calcein	(43, 44)
R175H, H179R, G245S, R248Q, R273H (overexpressed)	R175H, R273H	Ras-mediated signaling	BTG2, NF-κB^a^	CXCL1, IL1B and MMP3^a^	Human lung fibroblasts WI-38	–	–	(45)
R248Q, R249S, R273H (endogenous)	R175H, R248W	Chromatin epigenetic modification	ETS2^a^	MLL1, MLL2, MOZ^a^	Breast cancer cell lines, MEFs, Li–Fraumeni cell lines	Proliferation and tumor growth	COMPASS complex inhibitors	(27)

*^a^Transcription-related mediators and transcriptionally regulated downstream mutant p53 targets*.

Mutant p53 activities have been described both in the cell’s cytoplasm and in the nucleus. The reported cytoplasm-specific activities include the DAB2IP protein regulation affecting TNFα-dependent signaling (36) and the regulation of PARP localization and activity (38). Nuclear activities are related to more general influence on the chromatin function (the example of the above-mentioned MRE11 regulation) but, in most cases, are related to a specific transcriptional regulation. The available mutant p53 ChIP-sequencing data and other DNA-interaction data have not defined a mutant p53 target site analogous to that of wild-type p53, and currently the main hypothesis is that mutant p53 transactivation takes place through interaction with several transcription factors – among them NFY complex, SREBP 1 and 2, or ETS2 (Table 1). In most cases, mutant p53 proteins boost basic properties of these transcription factors, leading to the aberrant activation of their downstream programs and to the intersection with other key oncogenic pathways, as shown for the mutant p53-SREBP or NFY causing activation of the YAP/TAZ pathway (46, 47).

Even though experimental approaches using single initial *in vivo* and *in vitro* models led to the discovery of numerous pathways controlled by mutant p53, it is unclear whether these pathways have the same central role in diverse cellular contexts.

In an attempt to fill this gap, studies have been conducted involving the overexpression of multiple mutant p53 variants in a p53-null or wild-type background (Table 1). Investigation in the p53-null background of non-small lung carcinoma H1299 cells led to discovering the role of mutant p53 in integrin recycling (39), in the NF-κB signaling (40), and in the Warburg effect (41) as well as a role of TopBP1 in the upstream regulation of mutant p53 (43). These studies largely confirmed that the mutant p53 GOF is exerted indirectly at the level of transcription by cooperation with transcription factors. Neilsen et al. showed that genes activated by mutant p53 largely overlap between mutant variants overexpressed in H1299 cells, but interestingly also frequently share promoter sequences with p63 and wild-type p53 (42). This indicates that the mutant p53-mediated promoter activation may be an aberrant representation of the interaction of wild-type p53 with transcription factors in normal cells. Other mutant p53 overexpression studies led to uncovering regulation of the epithelial-to-mesenchymal transition (EMT) phenotype by mutant p53 upon wild-type *TP53* silencing in MCF10A mammary epithelium cells (48) as well as the cooperation of mutant p53 with the Ras oncogenic program in WI-38 human embryonic lung fibroblasts (45). Much of this evidence, however, still awaits confirmation in experimental settings in which mutant p53 variants are endogenously expressed. During the course of transformation, cell lines carrying endogenous TP53 mutations become addicted to the mutant p53 GOF – as often their growth or migration/invasion abilities are compromised upon mutant p53 knock-down (27, 31, 36, 49). Conversely, p53-null and wild-type p53 cell lines survive and proliferate without mutant p53, suggesting that very likely the GOF program observed under such conditions only partially resembles the cancer-related one. Therefore, the lack of the cellular context in which p53 mutants are naturally embedded and background-associated effects represent relevant weaknesses of the studies in a p53-null or p53 wild-type background.

A solution to these limitations may be represented by studies that include an initial analysis using different p53 mutants in their endogenous backgrounds. Analyzing downstream programs – both at the phenotypic and the molecular level – may help to understand to what extent p53 mutants possess a “core” oncogenic program, and whether some mutants display specific features. A recent study by Zhu et al. focuses on the common DNA interaction pattern of three distinct p53 mutants, in their endogenous context of breast cancer cell lines, using as term of comparison the pattern obtained from two cell lines bearing wild-type p53 (27). As a highlight of this multi-mutant p53–DNA interaction pattern, the group identified the chromatin regulatory genes that are activated by the transcription factor ETS2, a previously known mutant p53 interactor (23, 26, 35). The relevance of a mutant p53/ETS2 cooperation has been confirmed as a general feature in several mutant p53 expressing cell lines and thanks to the transcriptional program perturbed, as a critical modulator of the chromatin modification (27).

Even with these many studies published this is apparently only the beginning of a deeper understanding of both specificity and general picture of mutant p53 GOF in cancer. Multiple cellular/cancer models have to be studied simultaneously in unbiased, large-scale manner, by comparing more mutant p53 variants, including non-hotspot mutations. Another important issue is how these discoveries could be transferred into clinical applications.

## Targeting Mutant p53 in Cancer

The issue regarding how widely the GOF effects are shared between multiple mutant p53 variants extends to the experimental targeted therapies based on the presence of mutant p53. Since *TP53* is one of the most frequently mutated genes in cancer, reactivation of the wild-type p53 oncosuppressive properties and eliminating the mutant p53 GOF are potentially instrumental in personalized treatment of hundreds of thousands cancer patients worldwide. In this context, the possibility to distinguish mutant p53-specific processes from those shared by at least hotspot mutant p53 variants seems of relevance in order to develop and test drugs targeting properties and/or downstream pathways that are common to as many mutant p53 variants as possible.

Research on widely acting molecules targeting mutant p53 began over two decades ago. Some of the first approaches included inhibitors of Hsp90, a molecular chaperone that participates in a multiprotein complex stabilizing GOF p53 mutants with distorted DNA-binding domain structure (50). Hsp90 inhibitor geldanamycin was shown to lower levels and nuclear translocation of mutant p53 (51, 52). The interest toward Hsp90 inhibitors remains high, as the recent study by Alexandrova et al. describes significantly increased survival of mutant p53 KI mice treated with the geldanamycin derivative 17-DMAG or with a new generation Hsp90 inhibitor – ganetespib (53). Other drugs – such as the histone deacetylase inhibitor SAHA (Vorinostat) (54) and sodium butyrate (NaB) (55) – have been also shown to downregulate the levels of mutant p53 variants.

Different suggested strategies involve blocking the mutant p53 activation by targeting proteins, such as Pin1 (31) or TopBP1 (43). Inhibitor of TopBP1 – Calcein (44) – and experimental inhibitors of Pin1 (56) are examples of molecules targeting specific upstream activators of mutant p53. Among compounds that have been shown to efficiently directly target mutant p53 are small peptides (57–59). None of them, however, is so advanced in experimentation as small molecules that directly modify mutant p53 promoting its transition into a wild-type like form, capable of activating the tumor suppressive wild-type p53 transcriptional targets. The first described micromolecule targeting mutant p53 was CP-31398 (60) that, despite turning out not to directly interact with mutant p53 but rather with its target DNA sequences (58), is still considered as a promising drug candidate (61, 62). Most studies were, however, performed on the PRIMA-1 molecule (63) and later on its more potent and less toxic derivative PRIMA-1MET/APR-246 (64). Experiments showed that this molecule is able to directly bind and modify thiol residues in mutant p53 transforming it into a wild-type-like protein (65), thus becoming able to activate wild-type p53 targets, such as *GADD45B*, *NOXA*, or *CDKN1A* (p21), and induce *in vitro* and *in vivo* cell cycle arrest or apoptosis (66, 67).

In the case of drugs targeting mutant p53, most studied molecules, as those mentioned above, target several mutant p53 missense variants, while drug candidates focusing on particular mutants are rare. NSC319726 is one such these compounds. Identified by screening studies, NSC319726 possesses specific activity toward the R175H mutant p53 and induces apoptosis in human cells (68). Two other studies led to discovering PhiKan083 (69) and PK7088 (70) as molecules that specifically target and reactivate mutant p53 hotspot variant Y220C, which is found at a relatively high frequency in breast cancer (5). The low number of such studies and the fact that other mutant p53 reactivating compounds target various mutant p53 variants may suggest that the classic distinction of contact and structural p53 mutants may not be decisive and these mutant types may, in fact, represent structural extremes of a spectrum of distortions in the DNA-binding domain, leading to similar GOF effects.

**Figure 1:**
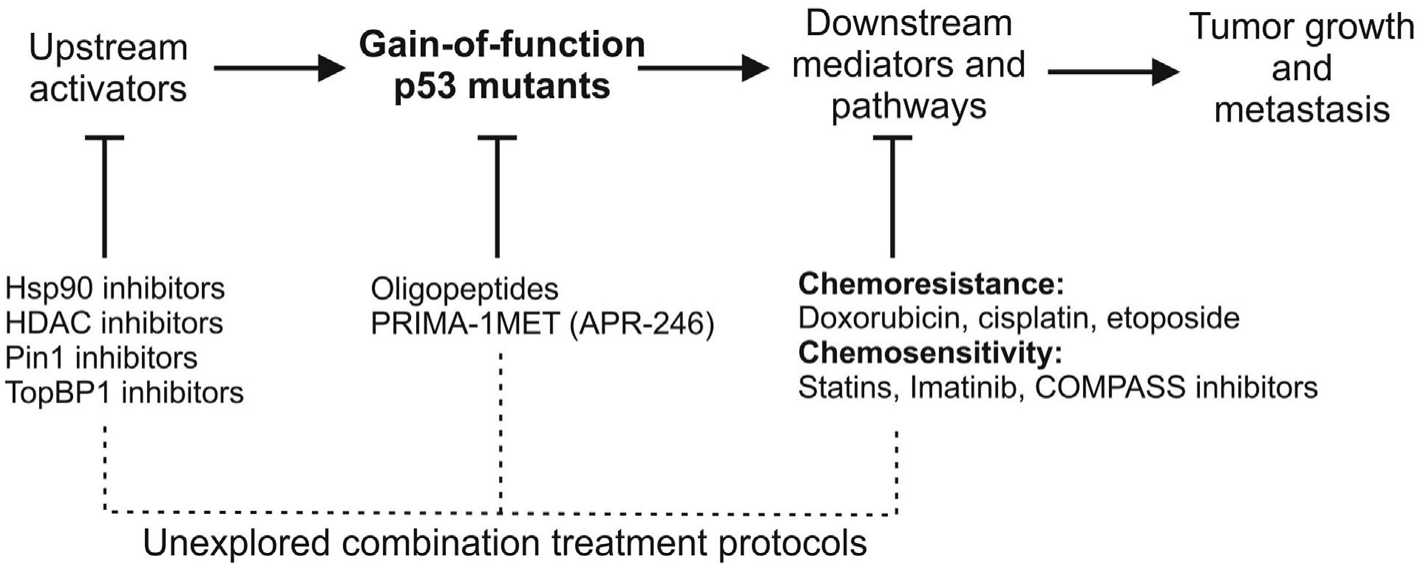
**A schematic view of gain-of-function mutant p53 activation, mutant p53 downstream effectors/pathways, and therapeutic opportunities of targeting each of the processes**. Below the largely unexplored possibilities of mutant p53-related combinational anti-cancer therapies are suggested.

Another important strategy to mutant p53 targeting is based on the treatment with drugs that downregulate oncogenic pathways activated by the means of mutant p53 GOF (listed in Table 1). This activation in general leads to two types of therapeutically relevant outcome – chemoresistance and chemosensitization (Figure 1). In the first case, the sensitivity to either specific or broad activity anti-cancer compounds, including doxorubicin, cisplatin, or etoposide, is dampened in the presence of mutant p53 (35, 49). In the latter case, a number of pathway targeting drugs – such as statins that inhibit the mevalonate pathway (33), imatinib inhibiting PDGFRβ (22), or COMAPSS complex inhibitors (27) – can cause increased cell death in mutant p53 vs. wild-type p53-bearing cancer cells. The performance of these drugs is often promising, but their drawback is the limited number of mutant p53/cell backgrounds tested.

A big issue of the mutant p53-oriented therapies is their slow progress toward the clinics, most of them being still at an early stage of development (71). The only drug directly targeting mutant p53 that has reached the clinical stage is PRIMA-1MET/APR-246. This compound successfully went through phase I/II clinical trial in hematological malignancies and prostate cancer that included mutant p53 patients (72). An approach targeting triple negative breast cancer (TNBC) cells with p53 deficiency or mutant status using Chk1 inhibitors showed promising results in *in vitro* and mouse tests (73, 74), while it failed to show significant improvement in human patients (75). At the same time, many of the drugs that could be beneficial for mutant p53 patients – Hsp90 inhibitors, HDAC inhibitors, or statins – are undergoing clinical trials in cancer in which the mutant p53 status is not considered or even known (76–78).

The combination of drugs directly targeting mutant p53 with drugs inhibiting mutant p53-related pathways is surprisingly avoided (Figure 1), although it might favor the decrease of compensatory responses and dosage toxicity, and thus an increase in the therapeutic efficacy. This notion is supported by a number of experiments showing that the combination of PRIMA-1 and PRIMA1-MET/APR-246 with cisplatin (CDDP) results in synergistic effects in cancer cells and xenografts (79–81). Taking into account that mutant p53 is known to increase chemoresistance to cisplatin (49), it is not surprising that targeting the cause of this chemoresistance opens the window to more effective treatments. This combinational approach may suggest that other compounds are worth being tested together with mutant p53 targeting drugs, such as PRIMA-1MET/APR-246 (Figure 1).

Even though *TP53* is one of the most frequently mutated genes in human cancer and mutant p53 emerges as a major oncoprotein controlling an exceptionally vast network of tumor-promoting activities, it still possesses underused potential as a drug target and much effort is needed to bring it to a prominent position on the map of personalized therapeutic solutions for cancer patients.

## Author Contributions

DW, KL, and GS wrote the paper.

## Conflict of Interest Statement

The authors declare that the research was conducted in the absence of any commercial or financial relationships that could be construed as a potential conflict of interest.
